# Microbiological diagnosis of pulmonary tuberculosis in children by oral swab polymerase chain reaction

**DOI:** 10.1038/s41598-019-47302-5

**Published:** 2019-07-25

**Authors:** Mark P. Nicol, Rachel C. Wood, Lesley Workman, Margaretha Prins, Cynthia Whitman, Yonas Ghebrekristos, Slindile Mbhele, Alaina Olson, Lisa E. Jones-Engel, Heather J. Zar, Gerard A. Cangelosi

**Affiliations:** 1 Division of Medical Microbiology and Institute for Infectious Diseases and Molecular Medicine, University of Cape Town, Cape Town, South Africa; 2 School of Biomedical Sciences, University of Western Australia, Perth, Australia; 3 Department of Environmental and Occupational Health Sciences, School of Public Health, University of Washington, Seattle, WA USA; 4 Department of Paediatrics and Child Health, and MRC Unit on Child & Adolescent Health, University of Cape Town and Red Cross War Memorial Children’s Hospital, Cape Town, South Africa; 5 Department of Anthropology, University of Washington, Seattle, WA USA

**Keywords:** Infectious-disease diagnostics, Tuberculosis

## Abstract

Microbiological diagnosis of pediatric pulmonary tuberculosis (TB) is challenging due to the difficulty of collecting and testing sputum from children. We investigated whether easily-obtained oral swab samples are useful alternatives or supplements to sputum. Oral swabs and induced sputum (IS) were collected from 201 South African children with suspected pulmonary TB. IS samples were tested by mycobacterial culture and Xpert MTB/RIF. Oral swabs were tested by PCR targeting IS6110. Children were categorized as Confirmed TB (microbiologic confirmation on IS), Unconfirmed TB (clinical diagnosis only), or Unlikely TB (recovery without TB treatment). Relative to Confirmed TB, PCR on two oral swabs per child was 43% sensitive and 93% specific. This sensitivity fell below that of sputum Xpert (64%). Among children with either Confirmed or Unconfirmed TB, PCR on two oral swabs per child was 31% sensitive and 93% specific, which was more sensitive than sputum testing among this group (21%). Although oral swab analysis had low sensitivity in sputum-positive children, it detected TB in a significant proportion of sputum-negative children who were clinically diagnosed with TB. Specificity at 93% was suboptimal but may improve with the use of automated methods. With further development, oral swabs may become useful supplements to sputum as samples for diagnosis of pulmonary TB in children.

## Introduction

Microbiological confirmation of pulmonary tuberculosis (TB) in children remains challenging despite recent advances^1^. Collection of specimens for testing, such as induced sputum (IS) or gastric aspirate, is perceived to be invasive and complex. Even when such specimens are obtained and tested, the diagnostic yield can be suboptimal, with microbiological confirmation achieved in only half of children diagnosed with pulmonary TB, even in well-resourced research studies^2^. Improved microbiological confirmation is important, not only for clinical care but also because assessment of novel diagnostic biomarkers for TB in children is severely constrained by the lack of a sensitive reference standard test.

One approach to improving TB diagnosis in children is to collect multiple different specimen types with the assumption that the yield would be cumulative. Potentially useful specimen types include gastric aspirates, induced sputum, nasopharyngeal aspirates, stool, urine, and the ‘string test’^3–6^. One of the simplest samples to obtain is a swab of the oral mucosa^7^. Originally demonstrated for diagnosis of TB in non-human primates^8,9^, we have shown that oral swab PCR could detect approximately 90% of microbiologically confirmed cases of pulmonary TB in adults^10,11^. As potential samples for pulmonary TB testing in young children, oral swabs (OS) have the advantage of being universally obtainable with minimal discomfort or need for operator training. We therefore investigated the diagnostic accuracy and incremental yield (over IS testing) of oral swab PCR for *Mycobacterium tuberculosis* DNA in children presenting to hospital with suspected pulmonary tuberculosis.

## Materials and Methods

In a prospective study, we enrolled 201 children (<15 years of age) presenting to Red Cross War Memorial Children’s Hospital, Cape Town, South Africa with suspected pulmonary TB between 13^th^ November 2016 and 22^nd^ September 2017. Enrolment criteria were cough of any duration plus one of: household contact with a TB source case within the preceding 3 months, loss of weight or failure to gain weight in the preceding 3 months, a positive tuberculin skin test (defined as ≥10 mm in HIV-uninfected children and ≥5 mm in HIV-infected children), or a chest radiograph suggestive of pulmonary TB. We excluded children if they had received more than 72 hours of TB treatment, were not resident in Cape Town, or if informed consent from a parent or guardian (and assent from older children) was not obtained.

Children provided two consecutive induced sputum specimens as previously described^12^. Following decontamination with sodium hydroxide, centrifuged sputum deposits were re-suspended in 1.5 ml of phosphate buffer. 0.5 ml of the re-suspended pellet was used for automated liquid culture (BACTEC MGIT, Becton Dickinson, Cockeysville, MD, USA). Positive cultures were identified by MTBDR*plus* testing (Hain Lifescience, Nehren, Germany). For the 1^st^ induced sputum, a further aliquot of 0.5 ml was used for Xpert MTB/RIF testing, as per manufacturer’s recommendations.

TB diagnostic categorization was based on clinical and microbiological investigations and follow-up visits, in line with revised NIH consensus definitions^13^: ‘Confirmed TB’ (any induced sputum culture or Xpert MTB/RIF positive for *M*. *tuberculosis*), ‘Unlikely TB’ (microbiologically negative, no TB treatment given and documented resolution of symptoms and signs at 3 month follow-up visit), or ‘Unconfirmed TB’ (all other children, including (i) children clinically diagnosed who were placed on TB treatment and (ii) children who were not placed on TB treatment and who had persistent symptoms and signs at follow-up).

Prior to sputum collection, two oral swabs (OS1 and OS2) were collected from each child, from the inside of the left cheek and the inside of the right cheek, respectively. In most cases the swabs were collected at the same time, one immediately after the other. In some cases they were collected up to 3 days apart. Swab collection always preceded sputum collection. GE Healthcare OmniSwabs or Puritan PurFlock swabs were used to scrape the buccal surface for 10 seconds. Swabs were immediately placed in a sterile, in-house lysis buffer^10^ for storage and frozen at −80 °C.

DNA was extracted from the swab samples by using a modified Qiagen protocol described previously^10,11^. Quantitative PCR targeting IS6110, a multi-copy insertion element unique to the *M*. *tuberculosis* complex, was done as described^11^. DNA extraction and qPCR were conducted in a 3-room laboratory suite with one-way workflow to minimize sample contamination. Laboratory-generated negative control swabs were extracted alongside sets of 10 OS samples and additional negative template controls were included in qPCR reactions. PCR results were categorized as positive or negative using a predetermined Cq cut-off of less than 40. The persons doing the PCR were blinded to reference standard test results and clinical information. Likewise, index test results and clinical information were not available to microbiology staff performing the reference standard testing, and the index test results were not available to the data analyst responsible for diagnostic categorization of children.

The attending physician, who made the decision whether to start TB therapy, had access to all laboratory results except the oral swab PCR. All enrolled children attended follow-up visits at 1- and 3-months post-enrolment. Children on TB treatment attended an additional follow-up visit at 6-months post-enrolment. Research staff assessed response to treatment at follow-up by recording symptoms, signs and weight gain. The Research Ethics Committee of the Faculty of Health Sciences, University of Cape Town approved the study. All methods were performed in accordance with the relevant guidelines and regulations. Informed consent was obtained through parents or guardians. No identifying information is presented in this report.

## Results

Of the 201 children enrolled with suspected pulmonary TB, 36 (18%) were excluded from further analysis for the following reasons: no second oral swab collected (n = 4), confirmed extrapulmonary TB only (n = 15), no valid induced sputum culture result (n = 11), or not started on TB treatment and did not return for follow-up assessment (n = 6). Of the 165 children included in the analysis, the median age was 31 (IQR 13–81) months, 87 (53%, 95% CI 45–61)) were female, 18 (11%, 95% CI 7–17) were HIV-infected, 84/137 (61%, 95% CI 53–70) had a positive TST, and 121 (73%, 95% CI 66–80) were started on TB treatment. Forty children (24%, 95% CI 18–32) were classified as confirmed TB, 81 (49%, 95% CI 41–57) as unconfirmed TB, and 44 (27%, 95% CI 20–34) as unlikely TB. The characteristics of the 165 children included in the analysis are detailed in Table 1. For 116 of the 165 children, the two swabs were collected at the same time, one immediately after the other. They were collected at different times for 49 of the children, ranging from 1 minute to 3 days apart. A valid Xpert MTB/RIF result from an IS sample was available in 154/165 (93%) children.

**Table 1:** Characteristics of children.

	All N = 165	Confirmed TB 40 (24.2%)^a^	Unconfirmed TB 81 (49.1%)	Unlikely TB 44 (26.7%)	P value
Age months Median (IQR)	30.5 (13.3–80.8)	23.0 (13.5–86.4)	24.2 (10.7–59.3)	45.2 (27.3–91.0)	0.122
Age category <5 years ≥5 years	113 (68.5) 52 (31.5)	27 (67.5) 13 (32.5)	61 (75.3) 20 (24.7)	25 (56.8) 19 (43.2)	0.103
Male sex	78 (47.3%)	15 (37.5%)	40 (49.4%)	23 (52.3%)	0.347
HIV infected	18 (10.9%)	4 (10.0%)	6 (7.4%)	8 (18.2%)	0.178
Started on TB treatment	121 (73.3%)	40 (100%)	81 (100%)	0 (0%)	<0.001
Tuberculin skin test positive^a^	84 (61.3%)	24 (75.0%)	52 (76.5%)	8 (21.6%)	<0.001

^a^Tuberculin skin test results available for a subset of the study population (N = 137).

We first calculated test accuracy (sensitivity and specificity) using children with Confirmed TB as the positive reference standard and those with Unlikely TB as the negative reference standard. As a secondary analysis we used a composite reference standard, combining the groups of Confirmed TB and Unconfirmed TB as the positive reference standard. We determined the accuracy of PCR on one oral swab (OS1, the first collected) and two oral swabs (OS1 and OS2, the first two collected). Reporting is as recommended by STARD guidelines for diagnostic accuracy studies. With an estimated proportion of children with Confirmed TB of 15%, this sample size was predicted to give 95% confidence intervals of 63–90% around a point estimate of 80% for oral swab PCR sensitivity.

Of the 165 children included in the analysis, 23 (14%) had a positive PCR on OS1, 27 (16%) had a positive PCR on OS2, and 39 (23%) had a positive PCR on either OS1 or OS2 (Table 2). Among those children with Confirmed TB, OS1 or OS2 were positive in 17/40 (43%). Among children with Unconfirmed TB, OS1 or OS2 were positive in 19/81 (24%). Among children with Unlikely TB, OS1 or OS2 were positive in 3/44 (7%). Oral swab positivity was significantly more frequent among children with Unconfirmed TB than among children with Unlikely TB (p = 0.020 by two-tailed z test).

**Table 2:** Number of positive tests, by TB diagnostic category and test type.

	Confirmed TB	Unconfirmed TB	Unlikely TB	Total
OS1^a^	12/40 (30%)	11/81 (14%)	0/44 (0%)	23/165 (14%)
OS2	12/40 (30%)	12/81 (15%)	3/44 (7%)	27/165 (16%)
OS1 or OS2	17/40 (43%)	19/81 (24%)	3/44 (7%)	39/165 (24%)
IS^b^ Xpert MTB/RIF^c^	23/36 (64%)	0/75 (0%)	0/43 (0%)	23/154 (15%)

^a^OS1, first oral swab PCR; OS2, second oral swab PCR.

^b^IS, induced sputum. Of 36 IS culture-positive subjects, 23 (64%) were positive on culture and Xpert, and 13 (36%) were positive on culture only.

^c^154 samples were tested by Xpert MTB/RIF.

Comparing OS results to Confirmed TB and Unlikely TB (Table 3), PCR on one oral swab (OS1) had a sensitivity of 30% (95% CI 17–47) and a specificity of 100% (95% CI 92–100). PCR on two oral swabs had a sensitivity of 43% (95% CI 27–59) and specificity of 93% (95% CI 81–99). For PCR on two OS, the positive likelihood ratio was 6.2 (95% CI 2.0–20) and the negative likelihood ratio was 0.6 (95% CI 0.5–0.8). By comparison, sensitivity of a single Xpert MTB/RIF test on induced sputum was 64% (95% CI 46–79). Although Confirmed TB was defined as any sputum culture-positive or Xpert MTB/RIF-positive for *M*. *tuberculosis* (per NIH consensus definitions), no Xpert MTB/RIF-positive, culture-negative cases were observed within the study population. Therefore, a secondary analysis was used to quantify the specificity of sputum Xpert MTB/RIF relative to sputum culture. This value was 100% (95% CI 92–100). The numbers of HIV-infected children were too small for assessment of HIV vs. non-HIV.

**Table 3:** Accuracy of one or two oral swab PCR tests when using two different positive reference standards and Unlikely TB as the negative reference standard.

Sensitivity reference standard	Index method	Sensitivity n (%) (95% CI)	Specificity n (%)^a^ (95% CI)	Positive LR^b^ (95% CI)	Negative LR (95% CI)
Confirmed TB	OS1^c^	12/40 (30) (17–47)	44/44 (100) (92–100)	NA^d^	0.7 (0.6–0.9)
Confirmed TB	OS1 OR OS2	17/40 (43) (27–59)	41/44 (93) (81–99)	6.2 (2–20)	0.6 (0.5–0.8)
Confirmed TB	IS^e^ Xpert MTB/RIF	23/36 (64) (46–79)	43/43 (100) (92–100)	NA	0.4 (0.2–0.6)
Confirmed + Unconfirmed TB	OS1	21/111 (19) (12–27)	43/43 (100) (92–100)	NA	0.8 (0.7–0.9)
Confirmed + Unconfirmed TB	OS1 OR OS2	34/111 (31) (22–40)	40/43 (93) (81–99)	4.4 (1–14)	0.8 (0.6–0.9)
Confirmed + Unconfirmed TB	IS Xpert MTB/RIF	23/111 (21) (14–29)	43/43 (100) (92–100)	NA	0.8 (0.7–0.9)

^a^Unlikely TB was the negative reference standard in all cases.

^b^LR, likelihood ratio.

^c^OS1, first oral swab PCR; OS2, second oral swab PCR.

^d^NA, not applicable. Positive LR could not be calculated when specificity was 100%.

^e^IS, induced sputum.

When OS positivity was compared to the combined group of Confirmed plus Unconfirmed TB (Table 3), in those 154 children with valid OS1, OS2, and induced sputum Xpert MTB/RIF results, the sensitivity of PCR on OS1 was 19% (21/111, 95% CI 12–27) and of PCR on OS1 or OS2 was 31% (34/111, 95% CI 22–40). By comparison, a single Xpert MTB/RIF test was positive in 21% (23/111, 95% CI 14–29) of children within this group.

## Discussion

Although PCR of oral swabs had low sensitivity for identification of children with culture-confirmed pulmonary TB, the method was positive in a substantial proportion of children who were clinically diagnosed with TB but had negative mycobacterial culture of induced sputum (Unconfirmed TB). This proportion was significantly greater that that seen in the Unlikely TB group, indicating that at least some of these children were likely to have had the disease. Therefore, while oral swab PCR is unlikely to replace testing of more traditional samples for diagnosing TB in children, it may have a useful complementary role.

This study focused on PCR testing of swabs, rather than bacteriological culture of swabs, because the former method yields results far more quickly. A limitation of this study was the use of a manual real-time PCR assay. This strategy was used in this proof-of-principle study because commercial sputum testing methods such as Xpert MTB/RIF are extensively engineered for sputum processing and may be suboptimal for swab analysis. However, manual PCR assays are prone to false-positive results due to laboratory cross-contamination. Two swabs were collected per child, so a total of 88 swabs were tested from 44 children in the Unlikely TB group. Of these, 3 were positive (3.4%). This false positivity rate was similar to that seen with “air swabs” tested by the same manual method in a previous study (3.6%)^11^. Therefore, at least some of the background signal seen in oral swabs may have resulted from contamination during manual analysis. We did not attempt to use sequencing or other methods to exclude the possibility of false-positive results arising from environmental exposure or non-pathogenic carriage. Overall, automation of oral swab methodology is needed to reduce false-positivity.

In addition, we compared the accuracy of OS PCR with that of liquid culture of a single induced sputum specimen. We, and others, have previously shown that there is substantial incremental yield from testing additional respiratory specimens^6,14^, and we therefore have likely over-estimated the sensitivity of OS PCR in relation to true disease status. Additional limitations included a small sample size and a focus on a single geographical area.

Given the challenges associated with diagnosing pulmonary TB in children, there is increasing interest in testing of additional sample types that are easily and universally obtainable from children. If oral swab testing is successfully adapted to cartridge-based nucleic acid amplification tests such as Xpert MTB/RIF Ultra, it may hold substantial promise in this regard.
